# Liveability and cities “in the middle”: an analysis of urban wellbeing from a foundational economy perspective

**DOI:** 10.3389/fsoc.2026.1890817

**Published:** 2026-10-07

**Authors:** Rossella Bozzon, Davide Arcidiacono, Maurizio Avola, Carlotta Ebbreo, Marcello Cabria

**Affiliations:** 1 Department of Political and Social Sciences, University of Bologna, Bologna, Italy; 2 Department of Political and Social Sciences, University of Catania, Catania, Italy; 3 Department of Political and Social Sciences, University of Cagliari, Cagliari, Italy

**Keywords:** foundational economy, Italian urban system, middle cities, territorial inequalities, urban liveability

## Abstract

This article examines liveability within the Italian urban system through the lens of the foundational economy. Existing approaches to liveability have increasingly relied on standardized and competitiveness-oriented frameworks, often overlooking the territorial and relational specificities of different urban contexts. Intermediate urban areas, despite their strategic role within national settlement systems, remain underexplored in current debates. To address this gap, the article focuses on cities “in the middle,” understood as medium-large urban centres occupying an intermediate position between large metropolitan hubs and marginal areas. Their middle condition refers to a specific socio-relational configuration shaped by demographic relevance, political-administrative centrality, service provision, and cultural and human-capital functions. Drawing on the foundational economy perspective, urban liveability is conceptualised as a multidimensional condition grounded in everyday life and articulated through three interconnected dimensions: foundational services, social infrastructure, and income. The empirical analysis develops a composite Liveability Index based on municipal-level indicators and applies it to 109 Italian provincial capital municipalities using ISTAT data. Results show that middle cities occupy a distinctive position within the Italian urban hierarchy, combining levels of foundational services and income comparable to those of larger urban centres but social infrastructure characteristics closer to those of smaller urban contexts. In particular, the social infrastructure dimension is a particularly discriminating dimension among cities in the middle but it is influenced by historical spatial divides between the northern and southern regions of the Country.

## 1 Introduction

The concept of liveability has been defined broadly and has changed over time. Originally connected to the Greek concept of *eudaimonia*, it assumed concrete importance in the 19th century with the growth of urbanization and industrial transformation processes. In particular, Dutch urban planning movements, led by the celebrated Housing Act of 1901 (*Woningwet*), had already redefined livability by linking it to centralized municipal planning and mass social housing. At the same time, proposals for redesigning urban space and addressing challenges to its livability (such as traffic, overcrowding, and pollution) emerged, such as Howard’s Garden City of 1898, which already addressed significant issues such as the creation of greenbelts, the centrality of mobility services, and the reduction of commuting, as well as the limitation of property speculation and real estate rents. Moreover, classical scholars have shown some interest in this issue: Simmel (1903, 1997) revealed how urban environments condition patterns of sociability, while Lefebvre (1968) articulated the “right to the city” as a claim to equitable access to space and services. During the 1920s and 1930s, sociographic schools (particularly in Austria, Poland, and Hungary) introduced empirical field research to scientifically map urban distress, such as the famous study on Marienthal, or the sociographic studies to support the “Red Vienna” model and the construction of large public housing complexes equipped with communal laundries, kindergartens, medical clinics, and green spaces. Thanks to these studies, livability was no longer understood solely as street hygiene but as a combination of social dignity, cultural accessibility, and welfare infrastructures. Similar reflections have also inspired the work of Jane Jacobs (1961). In her study, she did not use the word liveability, but her idea of the city based on a “chaotic” plurality to guarantee the quality of urban life without centralized direction or policies, is extremely relevant within this debate. From her perspective, accessibility and inclusivity are at the core of urban living, and they are linked to mixed-use neighborhoods that accommodate diverse functions, short blocks that foster social interaction and exchange, and sufficient density of people and housing to economically support commercial activities and services, as well as urban safety.

Nowadays, diverging from Jacobs’ perspective, the concept of liveability has become increasingly central to public and scientific debate. The evolution of the debate transformed liveability into a political device that frames and justifies urban planning and policymaking process (Ruth and Franklin, 2013). For example, the democratic U.S. party strongly used the context in the “Clinton Liveability Agenda” (1999) and in the Obama’s “Partnership for Sustainable Community” (2009). We can also mention international guidelines that aim to be strategies for national and international policy, politics, and economic development. For example in the World Banks’ “Livable cities for the 21st century” (World Bank, 1996) and “Keys to Cities” the urban liveability concept relates to accessibility to basic services, human and environmental health, as well as financial and productive cities; the United Nations Habitat Program with the “Urban Agenda” (from 2006 to 2016) identifies five action-areas to be planned for livable cities (National Urban Policy, Urban rules and regulations, Urban Planning and Design, Urban Economy and Municipal Finance, Planned City Extensions/Planned urban renewal); finally, the OECD “How’s Life in Your Region?” (2014) proposes approaches to measure regional and local wellbeing for policy making. Within this new perspective, by defining and measuring the relationship among city organization, quality of urban life, and socio-spatial inequalities (Paul and Sen, 2020), the concept has undergone a process of standardization of way of measurement (adopting indicators like on cycle paths, pollution indices, or smart technologies) that has accommodated competitive dynamics among cities and inspired highly data-driven policy approaches (Riepl et al., 2025). By this way, liveability measurement became more and more a normative process, and city-wide composite scores might obscure intra-urban inequalities and may privilege measurable amenities over lived experience, affordability, accessibility, social inclusion, or the needs of vulnerable groups. These approaches often overlook the dimensional and relational nature of urban contexts and their liveability, which instead requires a grounded perspective capable of recognising their territorial and historical-cultural specificities.

In this debate, Italy is a unique context, characterized by an urban environment of medium-sized and small cities that exhibit significant disparities in terms of development capacity and liveability. In this scenario, increasingly standardized studies on urban liveability fail to recognize the value of investigating the “middle” identity of Italian cities.

This article addresses this gap by focusing on the Italian case and, more specifically, on what we define as “cities in the middle”: medium-large cities that display social and cultural dynamism, yet are not fully integrated into major global economic flows or systemic networks of technological innovation. In the Italian urban system, these cities are particularly relevant because they often combine demographic relevance, territorial centrality, and service functions, without corresponding to large metropolitan hubs. Their “middle” status refers not merely to cardinal or ordinal magnitudes, nor to the formal classifications traditionally used in this field (mainly population size), but to specific socio-relational dimension of the city, rooted in its semi-peripheral condition (Hopkins and Wallerstein, 1982). This condition also shapes another specificity of these urban areas: the activation of coping strategies to gain space on the public agenda. Squeezed between large hubs and global attractors (Sassen, 2010; Perulli, 2012) and marginal or internal urban areas (Harrison and Heley, 2015; Barca et al., 2018), cities in the middle seek to redefine their role within the national and global urban system. Yet, despite their relevance, they remain underexplored in debates on urban liveability. In the empirical analysis, this intermediate position within the urban hierarchy is operationalised through the combination of demographic scale, service centrality, political-administrative function, and cultural/human-capital role.

Building on this gap, the article addresses the following research questions: Do middle cities in Italy display a distinctive liveability profile compared with other municipalities? Which dimensions of liveability account for this difference?

To answer these questions, the paper first reviews the literature on urban liveability and its measurement approaches, then shows how we develop the concept of cities “in the middle”, and frames liveability through the foundational economy perspective (Barbera et al., 2016; Calafati et al., 2023). Drawing on this framework, liveability is articulated into three pillars—foundational services, social infrastructure, and income—and operationalised through a composite index based on municipal-level indicators. The empirical analysis compares middle cities with other provincial capital municipalities in Italy.

## 2 The cities “in the middle” as a neglected issue for liveability

In recent decades, the debate on city and urban development has been strongly shaped by the idea of urban competitiveness. Cities have increasingly been framed as performative devices capable of reviving growth in developed economies affected by the post-Fordist crisis. This perspective has produced a functionalist simplification of the urban role in economic and social development, directing attention toward agglomeration processes, growth imperatives, and competitive positioning, while reducing attention to the social and environmental dimensions of urban life. Liveability has been treated as a by-product or asset of competitiveness rather than as an autonomous social, environmental, and distributive goal. Competitiveness discourse does not simply ignore liveability but often instrumentalise it, concentrating on the measurement of selected dimensions of liveability - amenities, connectivity, etc.—as crucial factors of urban attractiveness. For Florida (2002), the most competitive cities offer dynamic and stimulating urban environments that combine technological development with opportunities for leisure and spaces for interaction, particularly attractive for the so-called *creative class* (scientists, artists, programmers, managers). These elements represent also factors that can influence the location of large companies and turn into an investment multiplier. Glaeser (2012), on the other hand, sees how cities no longer compete as centers of material production (near ports or mines), but as places of consumption capable of offering high-level services and lifestyles. For this reason, this scholar still believes that liveability is the result of density, not an “abstract” phenomenon related to tolerance (as Florida argued) but grounded in the availability of essential services and housing. However, critical urban scholarship has shown that both these approaches fuel processes of selection and exclusion, as resources tend to concentrate in specific urban areas or clusters, while other urban contexts remain marginalised (Harvey, 1989; Soja, 2010; Pinson, 2020). Therefore, scholars and policy makers attention has focused mainly on large urban agglomerations and global cities, that is, those most integrated into global value chains and most central to economic and human flows (Sassen, 2010). Large cities are frequently represented as attractors of resources, opportunities, and social mobility (Michelangeli and Türk, 2019). In this view, liveability is associated with access to diversified services, dense networks of connections, and opportunities for enhancing human capital, even though these cities are also marked by increasing polarisation, environmental costs and socio-spatial inequalities.

At the opposite end of the urban hierarchy, marginal urban areas and small or medium-sized cities are often described through a different register. Here, liveability is associated less with access to global flows and more with dense social relations, community belonging and environmental quality, despite demographic decline, economic fragility, and reduced service provision (Barca et al., 2018). Between these two poles, however, the “middle” remains a relatively neglected analytical and relational dimension. The relationship between urban spaces, global and local competitive strategies, and citizens’ liveability has been much less explored in cities that do not fit either the model of the large competitive metropolis or that of marginal/internal areas.

The pandemic crisis, together with debates on proximity and the “15-min city,” has renewed attention to the dimensional and relational conditions of good urban living, emphasizing the interdependence between spaces, services, proximity and networks (Allam et al., 2022; Moreno et al., 2021; Manzini, 2021). From this perspective, medium and intermediate cities may be understood as potential hubs of liveability that deserve greater analytical scrutiny.

This issue is particularly relevant in Italy. Urban and regional studies have often neglected “middle” areas (Lanzani et al., 2021), despite the polycentric character of the European urban model (Bagnasco and Le Galès, 2000) and the relevance of intermediate urban centres within the Italian settlement system. These are semi-peripheral places: neither fully central nor marginal; neither fully dynamic nor stagnant. They are positioned between large cities capable of attracting resources and inhabitants, and marginal contexts from which they may themselves attract population, services and economic functions. Yet these cities may also experience demographic instability and economic fragility, without being explicitly recognised as marginal. As a result, they often fall outside dedicated policy attention at both national and international levels.

The lack of attention to cities “in the middle” has also had political and strategic consequences. While many global cities have developed long-term strategies for capitalizing on urban growth (Veldman, 2020), middle cities have often relied on forms of competitive isomorphism, through adoption of volatile or externally defined urban imaginaries and brands (Appadurai, 2013).

Our analytical focus is therefore on cities “in the middle”, a category that remains underexplored in debates on urban wellbeing. The concept is intended as an alternative to the conventional definition of medium-sized cities based exclusively on population size. Rather than identifying a purely demographic class, the notion of the middle cities refers to an analytically distinct category located between large metropolitan centres and small towns, combining demographic scale with political-administrative, economic, service-related, and cultural functions. In this sense, middle cities occupy an intermediate but strategic position within the urban hierarchy. They often act as important local hubs, providing essential services in areas such as trade, manufacturing, tourism, healthcare, education and transport, while serving catchment areas extending beyond their municipal boundaries. At the same time, they perform political and administrative functions and may display cultural and intellectual vitality linked to universities, higher education institutions, and research centres.

## 3 Liveability, meaning and measurement of a wide concept

Scholars agree that the concept of liveability is a buzzword without ashared definition, and its intrinsic vagueness bounces between political and academic discourses (Liang et al., 2020; Randhawa and Kumar, 2017). At the same time, the issue of urbanity in relation to liveability recognizes the ambivalent nature of cities that are at the same time wellbeing providers, for connectivity, services and goods accessibility, as well as providers of negative impacts on the environment and society, for pollution, traffic congestion, the fragmentation of social identities, and the increase of spatial and social inequalities (Zanella et al., 2015 Villanueva et al., 2015). Accessibility and inclusiveness in the urban environment are structurally rooted in space and in the socio-economic environment, as well as in the quality of political choices and infrastructure investments which are also influenced by historical and cultural legacies.

From this perspective, our study was preceded by a systematic literature review to assess how liveability has been analyzed, and whether and how the “middleness” dimension has been ever considered. Furthermore, we also wanted to assess the main dimensions considered from a measurement perspective.

Therefore, to conduct an in-depth exploration of the social science literature on *urban liveability*, we undertook an analysis of the publications indexed in Scopus.^1^ We identified 155 publications, with the earliest dating to 1999, however, the publication rate shows a marked increase beginning in 2015.

Most international studies in *urban studies* and *area studies*—are characterized by diverse analytical methodologies that frequently transcend disciplinary boundaries. Geographically, most of the research originates from Australia and the United Kingdom, while Italy ranks third in terms of publication output. The research topics examined represent a wide-ranging field that encompasses both subjective perceptions and normative considerations. They also address multiple scales of analysis—from the individual level to the local community (e.g., the neighborhood), to “status-based” communities (e.g., migrants, women, young families), and finally to the city in its various conceptualizations (e.g., the compact city, sustainable city, 15-min city, the metropolis). Simultaneously, this body of work considers the infrastructures—both material and immaterial—that shape urban environments, as well as the related reflections on urban planning and governance processes.

We align with Çalışkan and Şevik’s (2022) argument that the scholarly debate on liveability evolves along two main interpretative axes corresponding to its semantic foundations: *infrastructures* and *relations*. Table 1 shows as the two most cited documents emerging from the Scopus search define liveability through pillars of *cleanliness, health and conducive environments* (Allam and Dhunny, 2019) or adopting the Major Cities Unit’s definition (2010, p.7) where “liveable cities are socially inclusive, affordable, accessible, healthy, safe and resilient to the impacts of climate change. They have attractive built and natural environments. Liveable cities provide choice and opportunity for people to live their lives and raise their families to their fullest potential*”.* To further systematize the discussion while acknowledging the inherent overlaps, we propose thorough abstract and title analysis four thematic areas and related items—though not mutually exclusive—that capture the principal dimensions and approaches recurrent in the literature on urban liveability (Table 2). We identify four gazes of analysis (analytical dimensions), different analytical themes on which these perspectives are developed (main issues), and some reference studies for these approaches.

**Table 1:** Definition of liveability in the six more cited documents (1999–2024).

Reference	N. of citations	Main topic	Definition
Allam and Dhunny (2019)	819	How big data management can enhance liveability in smart cities	The paper mentions liveability under the threat of pollution and congestion, so, it refers to “*cleanliness, health and conducive environments*” as pillars of liveability and digital support (AI) as the essential tool to provide “responsive services both conveniently and in real time.” Moreover, to bridge efficiency and liveability in smart cities he uses Allam and Newman’s model (Allam and Newman, 2018) that bases city management on three interrelated elements: Metabolism, governance, and culture.
Badland et al. (2014)	224	How to merge urban liveability and social health determinants into indicators and measurements	The paper adopts the following definition: “*Liveable cities are socially inclusive, affordable, accessible, healthy, safe and resilient to the impacts of climate change. They have attractive built and natural environments. Liveable cities provide choice and opportunity for people to live their lives and raise their families to their fullest potential*” (Major Cities Unit, 2010).
Howley et al. (2009)	192	Determinants of the relation between high density and neighborhood liveability satisfaction	It uses the concept without an explicit and clear definition.
Villanueva et al. (2015)	127	How to develop spatial and environmental indicators to assess the contribution of public open spaces to urban health, wellbeing, and overall livability	This paper adopts the following definition: “*safe, attractive, socially cohesive and inclusive, and environmentally sustainable; with affordable and diverse housing linked to employment, education, public open space, local shops, health and community services, and leisure and cultural opportunities; via convenient public transport, walking and cycling infrastructure*” (Lowe et al., 2013, p.11).
Newton (2012)	106	How to co-relate liveability and sustainability	Liveability: relates to those attributes of a place, ranging in scale from dwelling and neighborhood to a city and its region that contribute to residents’ quality of life and wellbeing. As such, the concept embraces measures of residential amenity, human capital, social capital, human health, and the more qualitative elements of personal satisfaction and happiness.
Brilhante and Klaas (2018)	91	Desk analysis to evaluate the green city performance	Liveability is calculated through a set of indicators: Total Population; GDP per capita; Life Expectancy; Unemployment rate; Total internet Penetration; CO2 emissions; Green spaces per capita; population density; Length of mass transport network; Length of cycling lanes; shared of solid waste collected by the city; Access to potable water; Share of wastewater treated; Daily suspended particle levels

**Table 2:** Urban liveability dimensions ant key issue in the literature (1989–2025).

Analytical dimensions	Main issues	Main references
1. Socio-Demographic Dynamics	Demographic trends Subjective experiences and space-use conflicts Housing	Köberl et al. (2024), McCrea and Walters (2012) Macías Rodríguez et al. (2021), Fogelman and Christensen (2022)
2. Services and Infrastructures	Fundamental services Social infrastructures. Green infrastructures	Badland et al. (2015) Büyükcivelek et al. (2022) Collins et al. (2019) Büyükcivelek et al. (2022).
3. Planning and Governance	Planning and measurement methodologies Economic models	Cicerchia (1999) Hu (2015)
4. Innovation and Sustainability	Technological innovation Sustainability	Howley et al. (2009) Allam and Dhunny (2019)

In quantifying the prominence of these items within the literature, and being mindful of their intersections,^2^ our synthesis reveals that fundamental services constitute the more widespread research focus. Dsouza et al. (2023), based on a scoping review on liveability measures that resulted in 744 eligible studies, argue that “there was minimal consensus across studies on the conceptualization of livability. However, measured domains and indicators overlapped significantly. The topic of measurements, or indicators and methods for measuring liveability, is particularly interesting because it implies a normative process regarding what liveability means according to different schools of thought. Since it is a complex and composite concept, some studies have attempted to create composite indicators. Others have focused on calculating the weight of a specific variable on the perception of liveability. With respect to the first case, the creation of liveability indicators has played a central role in supporting the implementation of public policies. de Oliveira et al. (2023), while resuming liveability-indicators in academic literature, point out their importance in policy orientation. As they argue, earlier research has pointed out several difficulties, including the need for renewing indicators reflecting more the social and spatial inequalities that emerge within urban environments (Lowe et al., 2015; Chi and Mak, 2021), as well as the challenge of designing measures that remain valid in low- and middle-income countries, where demographic dynamics and urbanization patterns differ significantly from those of high-income ones (Chi and Mak, 2021). A wide range of indicators and quantitative metrics have been formulated to assess the multiple facets of liveability. For instance, the Global Liveability Index (GLI) evaluates quality of life in 173 cities worldwide based on dimensions such as culture, environment, healthcare, and education (The Economist Intelligence Unit, 2022). Valcárcel-Aguiar et al. (2019) developed a composite index of sustainable urban liveability for 58 Spanish cities, incorporating aspects such as consumption capacity, employment, safety, and infrastructure. Instead, the Urban Livability Index (Higgs et al., 2019) combines, through specific indicators, different axes of liveability: transport, social infrastructure, employment, walkability, housing, green infrastructure, and ambient environment (de Oliveira et al., 2023). Furthermore, there are various attempts to qualify indicators for specific contextual issues, as in the case of Madurai in India (Parvathy and Manonmani, 2025), which suffers particularly from socio-spatial differences due to overpopulation. The issue of health, as well as the relationship between transportation, walkability, and health, are among the main drivers often considered to be incidental to liveability,essential to its measurement. (Badland et al., 2015). The quantity and quality of public space were also highlighted in relation to urban liveability (Beck, 2009; Cheshmehzangi, 2015), as well as the quality, accessibility and quantity of residential opportunities (Lowe et al., 2020; Higgs et al., 2022). While the process used to validate the measures varied, most studies reported high levels of reliability and liveability seems correlated with similar measures (e.g., place satisfaction, neighborhood safety, and sense of place) and “self-reported health and wellbeing.” (Dsouza et al., 2023: 839). Last but not least, it is important to underline that few studies have related measurement methods to urban scales. Jaszczak et al. (2021) study analysis argue small towns tend to have restricted financial resources, lower investment capacity, and aging populations, which constrain the scope and ambition of revitalization projects. In the article “The urban dimension in spatial development: contributions from spatial economics”. Fratesi et al. (2025) treat city size not as a constraint but as a variable shaping economic performance and spatial interactions, emphasizing how density and diversity (including differences in skills, sectors, cultural backgrounds, and land uses) can drive urban vitality and liveability. Certainly, urban demography, in terms of population density and accessibility of services, is also a central issues in the most recent debates, such as about the *compact city* (Howley et al., 2009; Dessì, 2015) or within *the fifteen-minute city* (Moreno et al., 2021). Limiting the screening of the results to Italy, five main topics emerged from the cluster analysis of abstracts and titles (Urban planning approaches, space use conflicts and socio-spatial dimension of the city experience, public health, housing, environmental infrastructures). The larger number of studies focus on socio-spatial disparities relate to housing, through analysis in different urban systems (Vicari and Anselmi, 2020; Bottini and Costarelli, 2021, 2023; Alietti and Pujia, 2023; Bottini, 2024), underscoring the importance of the topic has in the national context and debate. D’Alessandro et al. (2017) underline public health as an essential topic to be included in liveability planning and analysis. More in general, in the Italian debate, we can list three schools that are important for the purpose of this study. The first, rooted in regional and urban economics, analyzes issues related to quality of life in urban contexts. For example, in the empirical study by Colombo et al. (2014), positive results emerge in medium and large cities in central and northern Italy, but considering only the demographic scale, while the full dimension of “middleness” is not taken into account. A second strand consists of studies in urban studies, urban geography, and regional planning, such as Secchi (2000, 2013), that emphasize the role of public space in contemporary cities, or Dematteis (1995) and Magnaghi (2000) for the focus on territorial relations, heritage, and local systems in where cities are included. Although these contributions do not treat both the concept of liveability and the category of “middleness” explicitly. The third strand concerns studies focused on medium-sized cities, conducted within the fields of urban geography and territorial policies. In this case, the focus is on the role of intermediate cities in the national settlement system, on polycentrism, on the provision of services, on administrative capacity, and on territorial attractiveness (Balducci et al., 2017). However, liveability is an empirical outcome of certain possible indicators studied rather than the object of analysis itself among these studies. Conversely, the Italian public debate is partly shaped by non-academic analysis on livability rankings, which, upon closer examination, not only frame liveability as an instrument of interregional competition but also expose many methodological limitations in their construction. For instance, the most widely cited ranking—the Quality of Life index published by Il Sole 24 Ore—produce a public discussion year-to-year about “rank shifts” but tends to underemphasize the extent to which indicators are periodically revised, and how certain administrative and demographic variables underpin the analysis. Certainly, the issue of influence on public debate is central to the foundational economy approach, which seeks to generate impact through interdisciplinary and methodologically rigorous analyses of livability Foundational Economy research programme frames livability as the outcome of household-level conditions shaped by residual income, access to essential services, and the availability of social infrastructure. This approach redefines local success in terms of the universal provision of a minimum yet comprehensive bundle of essential goods and services, an adequate level of residual income, and the enabling conditions for individuals and households to engage in political life and social networks (Foundational Economy Collective, 2012). In summary, the literature review indicates that the concept of *middleness*—whether understood in relation to urban typologies or in broader terms—has received limited attention in the international debate on livability. Moreover, the review confirms the absence of a shared framework for measuring livability. Moreover, the debate shows the importance given to basic infrastructures and basic services, as well as socio-ecological relations and subjective experience of the urban context. Finally, the Italian scholarly debate has not explicitly connected livability with the concept of *middleness*, neither has it significantly shaped the international discourse on the topic, at least according to the bibliometric criteria adopted in this review.

## 4 Research design and methods

### 4.1 Analytical framework and research question

Building on the gap identified in the previous pages, this section examines the position cities “in the middle” within the Italian urban system. As already argue, existing approaches to liveability have often relied on standardised measures and have paid limited attention to cities that occupy an intermediate position between large metropolitan centres and marginal areas. Against this background, the analysis combines the foundational economy perspective, an operational definition of middle cities, and a composite index of urban liveability. The aim is to assess whether middle cities in Italy display a distinctive liveability profile compared with other provincial capital municipalities, and to identify which dimensions account for this profile.

The theoretical point of departure is the foundational economy approach (Foundational Economy Collective, 2012). Drawing on Fernand Braudel's (1979) distinction between different spheres of economic life, this perspective does not conceive the economy (and, by extension, urban life) as a homogeneous domain governed by a single universal logic, such as profit maximization or capital return. Rather, it understands economic life as composed of distinct spaces that fulfill different needs and therefore require different regulatory principles, aligned with different orders of worth.

From this perspective, the foundational economy encompasses both “material” activities and distribution networks, such as water supply, housing, waste treatment and public transport, and “providential” activities, including healthcare, education, childcare and eldercare. These sectors constitute the essential infrastructure of everyday life and collective wellbeing. Their territorial embeddedness is not simply a desirable feature, but a constitutive one: the foundational economy is inherently rooted in places and settlement contexts. Based on these theoretical assumptions, urban liveability is understood as resting on three mutually connected pillars: foundational services, social infrastructure, and income (Figure 1).

**Figure 1:**
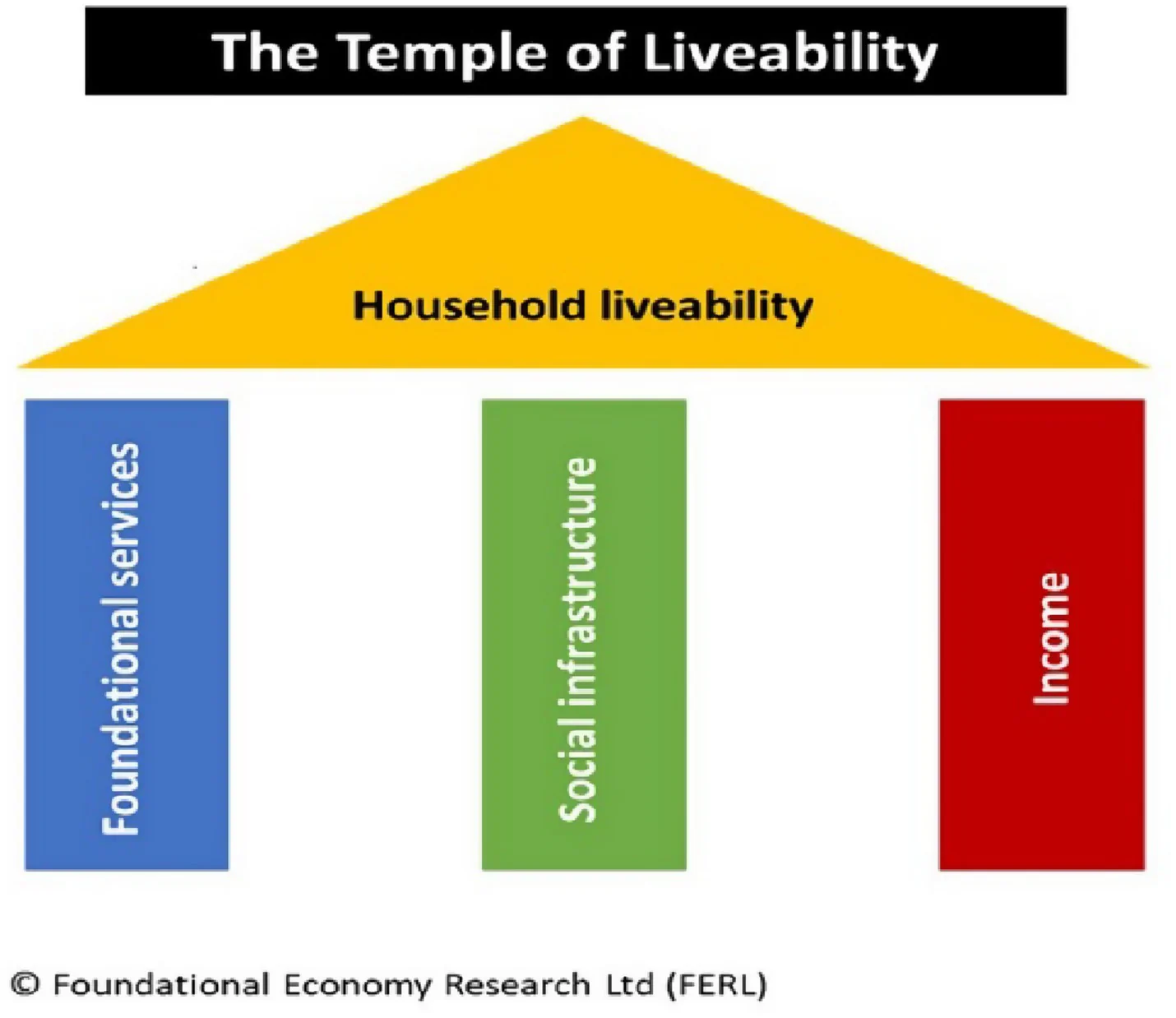
Liveability: main dimensions according to the foundational economy approach. Source: Calafati et al. (2023).

Foundational services are necessary for sustaining everyday life in contemporary urban societies, where the provision of essentials depends on complex socio-technical systems. They include welfare state services such as healthcare, education, and care, often provided free or charge or subsidised through taxation, but also utilities and housing, which are largely or entirely market-mediated and must be paid for out of household income. Therefore, income remains a crucial source of material wellbeing, but it cannot be considered independently of the availability and quality of foundational services.

Social infrastructure, in turn, refers to the set of free or affordable spaces and facilities locally available that enable sociability, participation and self-realisation (Renner et al., 2024). It supports the relational dimension of wellbeing, which extends beyond material sustenance to include interaction, belonging and community life.

### 4.2 Data and case selection

Building on the theoretical framework outlined above, the article develops a *Liveability Index* based on three analytical dimensions: foundational services, social infrastructure, and income. The index is operationalised through a set of municipal-level indicators selected according to theoretical relevance and data availability. Foundational services refer to the material and service infrastructures that sustain everyday urban life, including local welfare, early childhood services, water distribution, waste collection, public transport, and cycling infrastructure. Social infrastructure refers to the locally available facilities and resources that support sociability, participation, cultural access, and collective life, including libraries, museums, non-profit institutions, pedestrian areas, urban gardens, and urban green spaces. Income captures the economic and labour market conditions that allow households and individuals to access market-mediated goods and services that are not directly provided through collective or public infrastructures. The empirical basis relies on indicators available in the Istat municipal databases: “A misura di comune” information system^3^ and “Atlante Statistico del Territorio” (ASTer)^4^. The three dimensions capture complementary conditions of urban liveability from a foundational economy perspective. The full list of indicators, definitions, reference years and statistical treatments is reported in Table 3.

**Table 3:** Indicators and definitions.

Indicators	Definition/description	Year	Correction
FOUNDATIONAL SERVICES: Local welfare and care; basic material infrastructures, quality and reliability of network services, functioning of essential systems; mobility and daily accessibility			
Expenditure on social interventions and services	Municipal expenditure on social interventions and services/Resident population	2021	Log, normalization 0/1
Children aged 0–2 enrolled in municipal services	Children aged 0–2 benefiting from municipal early childhood services (nurseries, micro-nurseries, supplementary and innovative services)/Annual average resident population aged 0–2*100	2021	Log, normalization 0/1
Water losses in distribution (R)	Total water losses in the distribution system as a percentage of the volume introduced into the network *100 (* − 1)	2020	Reverse Log, normalization 0/1
Separate waste collection	Municipal waste collected separately/Total municipal waste collected *100	2021	Log, normalization 0/1
Seat-km supplied by local public transport	Kilometres actually travelled during the year by all local public transport vehicles * Average capacity of vehicles in operation/Average resident population	2021	Log, normalization 0/1
Overall demand for local public transport	Passengers per inhabitant	2021	Log, normalization 0/1
Density of cycle paths	Kilometres of cycle paths/Square kilometres of municipal area * 100	2021	Log, normalization 0/1
SOCIAL INFRASTRUCTURE: Cultural infrastructures; civil society infrastructures, and public and environmental spaces of sociality			
Libraries	Number of libraries/Resident population * 100,000 inhabitants	2021	Log, normalization 0/1
Museums, galleries, archaeological sites and monuments	Number of museums, galleries, archaeological sites and monuments/Resident population * 100,000 inhabitants	2020	Log, normalization 0/1
Libraries accessible to visitors with disabilities	Libraries with accessible entrances, spaces, facilities, services and dedicated workstations for visitors with disabilities/Resident population * 100,000 inhabitants	2021	Log, normalization 0/1
Museums accessible to children and persons with disabilities	Museums/institutions providing materials and dedicated routes for children and persons with disabilities/Resident population * 100,000 inhabitants	2020	Log, normalization 0/1
Number of non-profit institutions	Number of active non-profit institutions/Average resident population * 100,000	2020	Log, normalization 0/1
Availability of pedestrian areas	Square metres of pedestrian areas/Average resident population * 100	2019	Log, normalization 0/1
Urban gardens	Square metres of land allocated to urban gardens/Average resident population * 100	2021	Log, normalization 0/1
Urban green space	Square metres of urban green space/Average resident population * 100	2021	Log, normalization 0/1
INCOME: income inequality, employment and economic vulnerability, local economic structure			
Taxpayers with total income below EUR 10,000 (R)	Taxpayers with total IRPEF income below EUR 10,000/Total taxpayers * 100 (* − 1)	2021	normalization 0/1
Average taxable income	Taxable income/Taxpayers with taxable income	2021	normalization 0/1
Employment rate	Employed population aged 15 and over/Population aged 15 and over * 100	2021	normalization 0/1
Low work intensity of households (R)	Registered households with a work intensity below 20% of their potential/Total registered households * 100 (* − 1)	2019	normalization 0/1
Local unit density	Number of local units/Municipal area (sq km)	2021	Log, normalization 0/1

(R) indicates reverse-coded indicators.

Given the complexity of the index and the constraints imposed by data availability, the analysis is limited to provincial capital municipalities in 2021. This restriction reflects the fact that several indicators, especially those related to foundational services and social infrastructure, are systematically available only for these municipalities. Although this choice narrows the scope of the analysis, it ensures a more homogeneous and comparable empirical basis.

The focus on cities “in the middle” is operationalised through four criteria. Middle cities are defined as municipalities that simultaneously meet the following conditions: (i) a population between 45,000 and 500,000 inhabitants according to the 2021 Census; (ii) classification as a service hub according to the National Strategy for Inner Areas (SNAI) 2014–2020 framework; (iii) status as a political-administrative centre, that is, a provincial or metropolitan capital; and (iv) the presence of a university, excluding branch campuses, as an indicator of a cultural and human-capital provision function. The demographic thresholds were defined empirically by examining the relationship between municipal population size and the other variables used in the operational definition of cities “in the middle.” The lower threshold corresponds to the first population class in which more than half of municipalities are classified as service hubs and at least one quarter are provincial capitals. The upper threshold was set at 500,000 inhabitants, since above this level cities meet all the operational criteria considered and correspond to large metropolitan centres.

The decision to include only the *poles* (service hub), as defined by the Italian National Strategy for Inner Areas (SNAI), is based on the specific urban category under investigation. Although they are not major metropolitan areas, “cities in the middle” represent strategic urban centers within the Italian settlement system. This approach distinguishes them from the so-called “Italia di mezzo” (Lanzani, 2024), a concept used to describe territories in an intermediate position between poles and peripheral areas. According to the SNAI classification, poles are defined as centers endowed with the full range of essential services, whereas municipalities in the *belt areas* and beyond are characterized by lower accessibility to such services. The cities considered in this study, by contrast, are all classified as poles. Their “middleness” does not derive from an intermediate condition in terms of territorial marginality or service provision, but rather from their position between large metropolitan areas and smaller urban centers in terms of demographic size, institutional centrality, and urban, cultural and service functions. Within the empirical framework of the article, cities in the middle are compared with other provincial capital municipalities in Italy. In particular, the sample includes 109 municipalities, distributed amongfour groups: 38 middle cities; 48 other cities with populations between 45,000 and 500,000 inhabitants; 17 cities with fewer than 45,000 inhabitants; and 6 large cities. Their territorial distribution is reported in Table 4.

**Table 4:** Sample description: city type by geographical area.

	Middle cities	Other cities 45 k–500 k	Cities <45 k	Large cities	Total
North	16	20	8	3	47
Centre	6	12	3	1	22
South	16	16	6	2	40
Italy	38	48	17	6	109

This analytical choice also raises a methodological issue. By restricting the comparison to provincial capital municipalities, the empirical design selects cases that already satisfy, by definition, the political-administrative dimension and, in many cases, also the service dimension captured by the SNAI 2014–2020 classification. As a result, the analysis does not compare cities in the middle with the full range of Italian municipalities, but with a subset of territorially central urban centres. This does not invalidate the classification; rather, it clarifies its scope. The analysis should therefore be interpreted as assessing whether middle cities display a distinctive liveability profile within the group of political-administrative centres.

### 4.3 *Liveability index* construction

The *Liveability Index* is constructed as a formative composite measure grounded in the theoretical framework outlined in section 4.1. The construction procedure makes explicit the choices involved in indicator transformation, weighting, aggregation and robustness assessment.

All indicators are first harmonised and normalised on a common scale, ranging from 0 to 1. When appropriate, highly skewed indicators are log-transformed before normalisation to reduce the influence of extreme values. Indicators negatively associated with liveability are reverse-coded so that higher values consistently correspond to better liveability conditions.

Indicators are then aggregated to construct three sub-indices: the *Foundational Services Index*, the *Social Infrastructure Index*, and the *Income Index*. Within each dimension, indicators are combined through an equally weighted arithmetic mean, providing a transparent summary of each domain without imposing a finer hierarchy among its components. Equal weights are consistent with the foundational economy framework, which does not establish an explicit hierarchy among distinct components of urban liveability, such as childcare provision and public transport, cultural access and green space, or employment and income distribution. The implications of this choice are assessed through the robustness checks reported below.

The overall *Liveability Index* is computed using the geometric mean of the three sub-indices. The three dimensions are therefore treated as distinct but conceptually interconnected domains of urban wellbeing. Compared with the arithmetic mean, the geometric mean preserves equal nominal weights across dimensions while reducing the degree of linear compensation allowed among them. It therefore penalises unbalanced territorial profiles and partially limits the extent to which high performance in one domain can offset substantial weaknesses in another.

Descriptive statistics of the Liveability index and each sub-indices are reported in Appendix Table A1, and Table A2.

Several robustness checks were conducted to assess the sensitivity of each sub-indices to indicator selection and alternative weighting schemes, as well as the sensitivity of the overall *Liveability index* to alternative aggregation rules (OECD/European Union/EC-JRC, 2008).

Leave-one-indicator-out tests indicate that the rankings produced by each sub-index remain highly stable when indicators are excluded one at a time (Table A3a). Spearman’s rho remains above 0.972 for the Foundational Services Index, 0.954 for the Social Infrastructure Index, and 0.971 for the Income Index, showing that no single indicator drives the ranking structure.

Bounded Monte Carlo simulations further show that the alternative weighting schemes considered remain strongly correlated with the equal-weighted baseline (Table A3b). Even under the most demanding ±75% scenario, median Spearman correlations are very high: 0.983 for foundational services, 0.981 for social infrastructure, and 0.993 for income (Figure A1).

Principal Component Analysis (PCA) is used as a complementary exploratory diagnostic to assess covariance structure, shared directionality, and possible redundancy among indicators (Table A3c). The first principal component explains 45.5% of the variance for foundational services, 44.4% for social infrastructure, and 67.4% for income. This pattern is consistent with the greater homogeneity of the income dimension and with the more multidimensional character of foundational services and social infrastructure. All indicators load in the expected direction, supporting the directional coherence of the three sub-indices. At the same time, the variance structure suggests that the sub-indices cannot be reduced to single-factor reflective scales and is consistent with their interpretation as formative composite measures.

Taken together, the leave-one-indicator-out tests and Monte Carlo simulations support the stability of the equal-weighted sub-indices. The PCA diagnostic further suggests that the indicators move in the expected direction while retaining the multidimensional structure implied by the theoretical framework.

Robustness checks on the overall *Liveability index,* reported in Appendix Table A4, show that the results are highly stable across alternative aggregation rules. The baseline geometric specification is compared with an arithmetic mean, allowing full linear compensation, and with an harmonic mean, with a stronger penalty to low-performing dimensions. Spearman rank correlations, rank-shift analysis and quartile stability all indicate that the substantive ranking of cities is not sensitive to the chosen aggregation rule.

The empirical analysis proceeds in three steps. First, descriptive statistics are used to compare the distribution of the liveability index and its sub-indices across city types. Second, quantile regressions are estimated for the overall liveability index at the 25th, 50th and 75th percentiles. This strategy allows us to assess whether differences between city types emerge at different points of the liveability distribution rather than only at the mean. Third, the same models are estimated separately for the three sub-indices to identify which dimensions drive the relative position of middle cities. Considering the regional and supra-regional heterogeneity within the country (Avola, 2017; Asso, 2021), all models include geographical area as a control variable, distinguishing between North, Centre and South.^5^

## 5 Results

Figure 2 provides a descriptive overview of the distribution of the overall *Liveability Index* and its three sub-indices across city types. For the composite index, the boxplots suggest that cities in the middle occupy a relatively favorable position compared with cities with fewer than 45,000 inhabitants and with other cities with populations between 45,000 and 500,000 inhabitants. At the same time, their values are broadly comparable to those of large cities.

**Figure 2:**
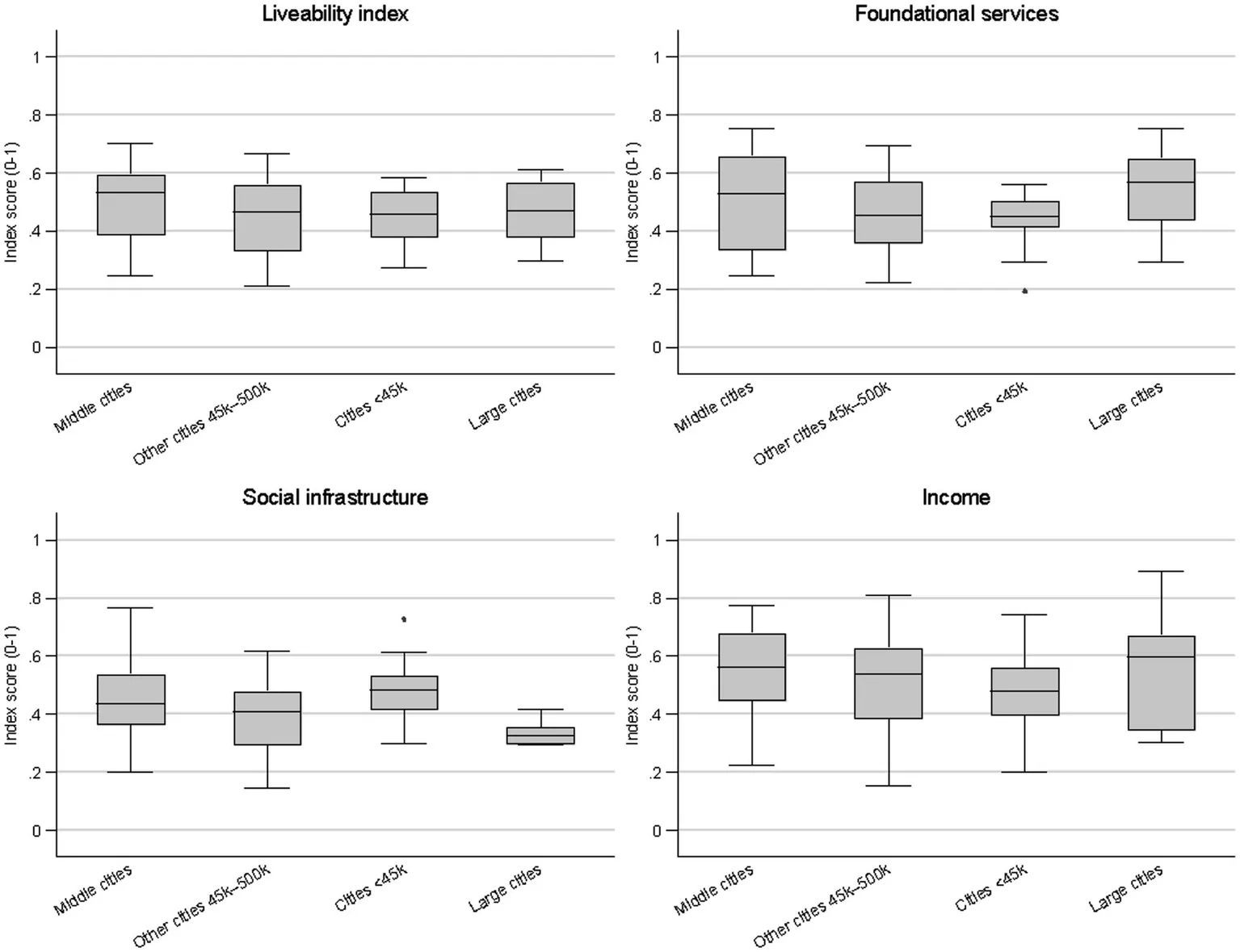
Distribution of liveability index, foundational services index, social infrastructure index, and income index by city type.

The three sub-indices reveal a more differentiated pattern. For the *Foundational Services Index,* cities in the middle appear closer to large cities, with both groups generally showing higher values than smaller cities and other cities in the same demographic range. A similar pattern emerges for the *Income Index,* where middle cities are again positioned closer to large cities than to the other two groups. By contrast, for the *Social Infrastructure Index*, middle cities display a distribution closer to that of cities with fewer than 45,000 inhabitants, while large cities appear to occupy a less favorable position. This pattern is consistent with middle cities displaying some of the relational and collective features usually associated with smaller urban contexts. Overall, the descriptive evidence points to a hybrid profile of the cities in the middle: they combine foundational services and income levels closer to those of larger urban centres, with social infrastructure levels relatively stronger and more like those of smaller cities.

The quantile regression results reported in Table 5 refine this descriptive picture and are consistent with the interpretation of cities in the middle as a distinctive urban configuration. After controlling for geographical area, other cities with populations between 45,000 and 500,000 inhabitants show lower scores than middle cities across all three quantiles. The estimated coefficients range from approximately −0.080 at Q25 to −0.062 at Q75. This suggests that, within the group of provincial capitals, cities in the middle tend to display higher values on the overall *Liveability Index* than other cities of comparable demographic size.

**Table 5:** Quantile regression estimates for the Liveability Index.

	Q25	Q50	Q75
City type			
Middle cities	Ref.	Ref	Ref
Other cities 45 k-500 k	−0.080**	−0.068**	−0.062*
Cities <45 k	−0.053	−0.068*	−0.051
Large cities	−0.019	−0.058	−0.030
Geographical area			
Reference: North	Ref.	Ref	Ref
Center	−0.098**	−0.066*	−0.066*
South	−0.259**	−0.234**	−0.234**
Constant	0.576**	0.608**	0.642**
Observations	109	109	109
Pseudo *R*^2^	0.490	0.483	0.417

**p < 0.01, *p < 0.05.

The results for smaller provincial capitals are more nuanced. Cities with fewer than 45,000 inhabitants show significantly lower scores than middle cities only at the median, while differences at the lower and upper quantiles are not statistically significant. Large cities do not show statistically significant differences from middle cities at any quantile. This suggests that, within the group of provincial capitals, large cities do not appear to differ systematically from cities in the middle in terms of overall liveability.

Geographical area is also strongly associated with liveability scores. Compared with cities in the North, cities in the Centre and especially in the South show significantly low scores across all quantiles. The coefficient for the South is particularly large and stable, and statistically significant across all three quantiles, while the Centre shows a more moderate but still significant disadvantage. These results are consistent with the relevance of Italy’s territorial divide, while suggesting that the relative position of middle cities remains visible when broad geographical areas are included in the model.

Overall, Table 5 is consistent with interpreting middle cities not merely as a demographic category, but as a specific urban configuration. Their relatively favourable position is especially visible when they are compared with other cities of similar size, suggesting that population size alone does not fully capture differences in liveability among provincial capitals. Robustness checks reported in Appendix Table A5, show that this substantive pattern is broadly stable when the *Liveability Index* is computed using alternative aggregation rules.

The analysis of the three sub-indices, reported in Table 6, further clarifies the relative position of middle cities. Since middle cities are used as the reference category, negative and significant coefficients for the other groups identify the dimensions in which middle cities show higher relative scores.

**Table 6:** Quantile regression estimates for the three Liveability sub-indices.

	Foundational services index			Social infrastructure index			Income index		
	Q25	Q50	Q75	Q25	Q50	Q75	Q25	Q50	Q75
City type									
Middle cities	Ref.	Ref.	Ref.	Ref.	Ref.	Ref.	Ref.	Ref.	Ref.
Other cities 45 k–500 k	−0.025	−0.051	−0.089**	−0.060	−0.068*	−0.051	−0.056*	−0.075*	−0.071*
Cities <45 k	−0.110*	−0.116**	−0.106	0.017	0.016	0.034	−0.093**	−0.093*	−0.107**
Large cities	−0.012	0.022	0.006	−0.112*	−0.099	−0.119*	0.010	−0.029	−0.033
Geographical area									
North	Ref.	Ref.	Ref.	Ref.	Ref.	Ref.	Ref.	Ref.	Ref.
Center	−0.140**	−0.110**	−0.111**	−0.046	−0.035	0.033	−0.047	−0.108**	−0.100**
South	−0.255**	−0.246**	−0.240**	−0.196**	−0.154**	−0.154**	−0.304**	−0.334**	−0.268**
Constant	0.559**	0.629**	0.710**	0.453**	0.516**	0.569**	0.597**	0.700**	0.763**
Observations	109	109	109	109	109	109	109	109	109
Pseudo *R*^2^	0.378	0.408	0.410	0.278	0.238	0.239	0.539	0.449	0.417

**p < 0.01, *p < 0.05.

For the *Foundational Services Index*, the relative position of middle cities is selective. Cities with fewer than 45,000 inhabitants show significantly lower scores at the lower and median quantiles, while other cities with populations between 45,000 and 500,000 inhabitants show lower scores at the upper quantile. Large cities, however, do not differ significantly from middle cities. This pattern suggests that middle cities are closer to large cities than to smaller ones in terms of foundational services.

For the *Social Infrastructure Index*, the pattern is different. Cities with fewer than 45,000 inhabitants do not show statistically significant differences from middle cities, whereas large cities show significantly lower values at the lower and upper quantiles. This result is a particularly relevant because it suggests that middle cities display a social infrastructure profile closer to that of smaller cities, while showing higher values than large cities in parts of the distribution. Other cities with populations between 45,000 and 500,000 inhabitants also show lower values at the median.

For the *Income Index*, middle cities again occupy a relatively favourable position. Other cities with populations between 45,000 and 500,000 inhabitants show lower scores than middle cities across the distribution, while cities with fewer than 45,000 inhabitants are also significantly lower. Large cities, by contrast, do not differ significantly from middle cities. This pattern suggests that middle cities share with large cities a relatively stronger socio-economic profile, while showing higher *Income index* values than smaller cities and other similarly sized cities.

The territorial pattern is also visible across the three sub-indices. Southern cities show consistently lower scores than Northern cities, with particularly large differences in income and foundational services. The Centre shows a more selective difference, mainly in foundational services and income, while no statistically significant differences emerge for social infrastructure. These findings are consistent with the idea that liveability differences among Italian provincial capitals are embedded in broader territorial inequalities, although these inequalities do not fully absorb the observed profile of middle cities.

Overall, Table 6 suggests that the relative position of middle cities is not based on uniformly higher values across all dimensions. Rather, their observed profile combines features usually associated with different urban scales: social infrastructure levels comparable to those of smaller cities, and foundational services and income levels comparable to those of large cities.

## 6 Conclusion

This article has examined urban liveability within the Italian urban system through the lens of the foundational economy (Foundational Economy Collective, 2012). Measuring urban liveability has become increasingly central to the debate, even though the conceptual boundaries of the phenomenon remain indeterminate and shifting. Furthermore, its increasingly emerging role as a policy device implies paying greater attention to how liveability is conceived and measured.

Our analysis clearly shows that many of the measurement systems adopted are designed with a functionalist perspective, reproducing in a simplified manner the issue of territorial development and wellbeing in a competitive context among cities, outlining isomorphic trajectories inspired by the model of large, more attractive global centers. Rather than treating liveability as a standardised measure of urban performance, we have adopted it as a multidimensional condition rooted in everyday life, shaped by access to foundational services, social infrastructure, and income.

In particular, the article focuses on cities “in the middle”, medium-large urban centres in intermediate position between large metropolitan hubs and marginal areas. Their middle condition refers to a specific socio-relational configuration shaped by demographic relevance (a population between 45,000 and 500,000 inhabitants), political-administrative centrality (status of provincial or metropolitan capital), service provision (status of hub according to the National Strategy for Inner Areas), and cultural and human-capital functions (presence of a university). These cities show the highest level of liveability compared to the other urban configurations taken into account (large cities, between 45,000 and 500,000 inhabitants, cities below 45,000 inhabitants). However, the most important finding of our study is that cities “in the middle” represent a distinct urban configuration: they do not outperform all other city types across every dimension of liveability considered, but display a specific profile, retaining levels of social infrastructure close to those of smaller cities, while showing foundational services and income levels closer to those of large cities. Re-focusing attention on the relevance of the “middle” dimension and the specificities of contexts (Chi and Mak, 2021) has proven to be a fruitful analytical path, capable of enhancing the quality of contexts other than those of large global attractors. At the same time, focusing on the “foundational” dimensions of liveability (Calafati et al., 2023) means regaining attention on the social dimension rather than the performative dimension of urban environments. The findings of our study support the idea that middle cities represent a garrison of liveability thanks to the distinctive characteristics that characterize them and to the greater accessibility and availability of infrastructures and services, where economic inequalities are more contained, and distributive conflicts less strong. This combination points to a hybrid profile of cities “in the middle” and helps to explain their favourable position in the Liveability Index. This result matters because it shifts attention away from a simple opposition between large metropolitan centres (Sassen, 2010; Perulli, 2012) and marginal areas (Harrison and Heley, 2015; Barca et al., 2018). Cities “in the middle” occupy a more complex position: they are not global hubs, but neither are they peripheral places. They are urban centres where material provision, social relations and economic conditions intersect in specific ways. In this sense, the foundational economy perspective helps capture what is often missed by more standardised approaches to liveability: the value of ordinary infrastructures and social conditions that make urban life of possible. The social and service dimension of liveability are more necessary than ever if we also consider liveability as a policy tool capable of guiding urban governance and management strategies. As our data clearly shows, social infrastructure is the particularly discriminating dimension across cities (Renner et al., 2024), even within “middle” cities. Furthermore, this dimension is particularly relevant in highlighting the persistence of historical gaps between cities in the North and those in the South. Our study confirms that liveability remains strongly shaped by the broader territorial inequalities characterizing the country (Avola, 2017; Asso, 2021), with a persistent disadvantage of Southern cities. However, the potential of cities in the middle respect to other urban configurations remains valid beyond their geographical location.The article also has some limitations.

The analysis is restricted to provincial capital municipalities because of data availability, and the composite index inevitably simplifies the complexity of liveability. Future research could extend the analysis to a wider set of municipalities, examine change over time, and explore more closely how local governance and institutional capacity shape the relationship between foundational provision and urban wellbeing, also from a more qualitative and ethnographic perspective.

## Data Availability

The original contributions presented in the study are included in the article/supplementary material, further inquiries can be directed to the corresponding author.

## Footnotes

1 The study initially aimed to synthesize the literature on urban liveability published between 1999 and 2025, adopting the same time frame as the empirical research underpinning this theoretical work. The initial search yielded 1,120 publications. A keyword-based screening was then conducted, retaining only documents explicitly referring to the terms *livability* and *quality of life*. This step served both to clarify the conceptualization of liveability and to align the review with the subtopics relevant to the study and its fundamental economics perspective. To ensure consistency, only books, book chapters, and journal articles were included, while other document types were excluded. The review was further restricted to the social sciences to engage with the debate most relevant to the research framework, excluding contributions rooted primarily in the natural sciences or disciplines such as informatics, psychology, and medical sciences. Following the removal of three duplicates, the titles and abstracts of the remaining 155 publications were screened and organized by 12 codes, which corresponded to the primary areas of analysis in the studies under consideration. There, we mainly focus the text analysis on documents coded as planning and measurement methods, literature review, and demographic related issues (85), excluding documents with a gaze of analysis only partially related to our research such as the ones focusing specifically on mobility (26), particular subjectivities’ experience of livability (16), particular city users conflicts (10), green infrastructures (9), social infrastructures (13), health infrastructures (9), or mainly on ecological sustainability issues (2). Within this group of 155, a second screening of abstracts and contents was performed on the results of studies limited to Italy (21), and one study was excluded because not relevant to the study (centred in a political science analysis of election dynamics). In addition to the search on Scopus, an in-depth analysis of Italian literature has been added to the literature review.

2 In fact, these themes often overlap and are therefore not mutually exclusive; rather than focusing on their quantification, we concentrate on identifying the main thematic lines and guiding approaches.

3
https://www.istat.it/statistica-sperimentale/aggiornamento-degli-indicatori-del-sistema-informativo-a-misura-di-comune/

4
https://www.istat.it/sistema-informativo-6/atlante-statistico-del-territorio/

5 This threefold classification was preferred to more fine-grained geographical partitions for methodological reasons. Given the small sample size and the uneven distribution of city types across territorial areas (Table 4), a fivefold classification would have generated very small cell counts in some groups, reducing the stability of the estimates. The North–Centre–South distinction therefore provides a parsimonious control for Italy’s main territorial cleavage while preserving adequate model robustness.
