# Supplementary material for: Liveability and cities “in the middle”: an analysis of urban wellbeing from a foundational economy perspective
Source: Front Sociol. 2026 Oct 7;11:1890817. doi: 10.3389/fsoc.2026.1890817 (PMC13689568; doi:10.3389/fsoc.2026.1890817)
Supplement: Supplementary file 1 [file Data_Sheet_1.docx]

**Appendix**

| **Table A1 - Liveability Index (0-1 scale) by city type. Descriptinve statistics (N.=109 cities)** | | | | | | | |
| --- | --- | --- | --- | --- | --- | --- | --- |
|  | **Mean** | **s.d.** | **Min.**  **value** | **25th**  **pct** | **Median** | **75th**  **pct** | **Max.**  **value** |
| **City type** |  |  |  |  |  |  |  |
| Middle cities | 0.496 | 0.137 | 0.247 | 0.386 | 0.534 | 0.596 | 0.702 |
| Other cities 45k–500k | 0.444 | 0.128 | 0.210 | 0.330 | 0.467 | 0.559 | 0.666 |
| Cities <45k | 0.458 | 0.095 | 0.275 | 0.379 | 0.457 | 0.534 | 0.582 |
| Large cities | 0.466 | 0.117 | 0.298 | 0.378 | 0.470 | 0.568 | 0.613 |
| *Total* | *0.466* | *0.127* | *0.210* | *0.376* | *0.483* | *0.572* | *0.702* |
|  | | | | | | | |

**Table A2 - Liveability sub-indexes (0-1 scale) by city type. Descriptive statistics (N=109 cities)**

|  | Mean | 25^th^  percentile | Median | 75th  percentile |
| --- | --- | --- | --- | --- |
| **Foundational services index** | | | | |
| **City type** |  |  |  |  |
| Middle cities | 0.509 | 0.333 | 0.528 | 0.658 |
| Other cities 45k–500k | 0.460 | 0.356 | 0.454 | 0.570 |
| Cities <45k | 0.436 | 0.411 | 0.450 | 0.503 |
| Large cities | 0.545 | 0.436 | 0.568 | 0.651 |
| *Total* | *0.478* | *0.363* | *0.477* | *0.585* |
| **Social infrastructure index** | | | | |
| **City type** |  |  |  |  |
| Middle cities | 0.447 | 0.362 | 0.437 | 0.538 |
| Other cities 45k–500k | 0.390 | 0.294 | 0.41 | 0.480 |
| Cities <45k | 0.480 | 0.415 | 0.482 | 0.532 |
| Large cities | 0.336 | 0.296 | 0.327 | 0.355 |
| *Total* | *0.421* | *0.346* | *0.418* | *0.513* |
| **Income index** | | | | |
| **City type** |  |  |  |  |
| Middle cities | 0.546 | 0.444 | 0.564 | 0.680 |
| Other cities 45k–500k | 0.502 | 0.384 | 0.538 | 0.627 |
| Cities <45k | 0.472 | 0.395 | 0.478 | 0.560 |
| Large cities | 0.568 | 0.346 | 0.5997 | 0.671 |
| *Total* | *0.516* | *0.426* | *0.537* | *0.650* |

**Tables A3 report robustness checks for the three formative sub-indices. Leave-one-indicator-out tests assess sensitivity to indicator selection (Table A3a), bounded Monte Carlo simulations assess sensitivity to alternative internal weighting schemes (Table3b and Figure A1), and PCA provides an exploratory data-driven benchmark (Table 3c).**

**Table A3a Leave-One-Indicator-Out**

| **Sub-index** | **Excluded Indicator** | **Spearman’s rho** | **Kendall’s tau** |
| --- | --- | --- | --- |
| **Foundational services index** | Density of cycle paths | 0.972 | 0.859 |
|  | Water losses in distribution (R) | 0.973 | 0.857 |
|  | Separate waste collection | 0.978 | 0.880 |
|  | Children aged 0–2 enrolled in municipal services | 0.983 | 0.901 |
|  | Seat-km supplied by local public transport | 0.984 | 0.898 |
|  | Overall demand for local public transport | 0.984 | 0.896 |
|  | Expenditure on social interventions and services | 0.994 | 0.948 |
| **Social infrastructures index** | Urban gardens | 0.954 | 0.820 |
|  | Availability of pedestrian areas | 0.983 | 0.898 |
|  | Urban green space | 0.984 | 0.905 |
|  | Museums accessible to children and persons with disabilities | 0.984 | 0.904 |
|  | Number of non-profit institutions | 0.985 | 0.899 |
|  | Libraries accessible to visitors with disabilities | 0.987 | 0.923 |
|  | Museums, galleries, archaeological sites and monuments | 0.990 | 0.919 |
|  | Libraries | 0.991 | 0.925 |
| **Income index** | Local unit density | 0.971 | 0.852 |
|  | Low work intensity of households (R) | 0.983 | 0.891 |
|  | Employment rate | 0.990 | 0.919 |
|  | Taxpayers with total income below EUR 10,000 (R) | 0.991 | 0.926 |
|  | Average taxable income | 0.995 | 0.945 |

**Table A3b. Monte Carlo bounded random-weight sensitivity analysisMonte Carlo bounded random weights. Scenarios: +/-25%, +/-50%, +/-75%**

|  |  | **Spearman’s rho** | | | **Kendall’s tau** | | |  |  |
| --- | --- | --- | --- | --- | --- | --- | --- | --- | --- |
| **Subindex** | **Bound** | **Min** | **Median** | **Max** | **Min** | **Median** | **Max** | **Replications** | **N Indicators** |
| **Foundational services index** | +/-25% | 0.992 | 0.998 | 1.000 | 0.926 | 0.965 | 0.994 | 5000 | 7 |
|  | +/-50% | 0.970 | 0.992 | 1.000 | 0.847 | 0.929 | 0.992 | 5000 | 7 |
|  | +/-75% | 0.921 | 0.983 | 0.999 | 0.750 | 0.891 | 0.982 | 5000 | 7 |
| **Social Infrastructure index** | +/-25% | 0.988 | 0.997 | 1.000 | 0.917 | 0.963 | 0.992 | 5000 | 8 |
|  | +/-50% | 0.962 | 0.991 | 0.999 | 0.844 | 0.927 | 0.983 | 5000 | 8 |
|  | +/-75% | 0.892 | 0.981 | 0.999 | 0.722 | 0.891 | 0.978 | 5000 | 8 |
| **Income index** | +/-25% | 0.993 | 0.999 | 1.000 | 0.934 | 0.978 | 0.999 | 5000 | 5 |
|  | +/-50% | 0.966 | 0.996 | 1.000 | 0.846 | 0.955 | 0.999 | 5000 | 5 |
|  | +/-75% | 0.899 | 0.993 | 1.000 | 0.730 | 0.932 | 0.995 | 5000 | 5 |

Note: For each replication, indicator weights were randomly perturbed around the equal-weight baseline within the specified bounds and then normalised to sum to one. Each simulated sub-index was compared with the baseline equal-weighted sub-index using Spearman’s rho and Kendall’s tau. Each scenario is based on 5,000 replications.

**Table A3c. Principal Component Analysis (PCA) results for sub-indices**

| Dimension | No. indicators | N | Eigenvalue PC1 | Variance explained PC1 (%) | Variance explained PC1–PC2 (%) |
| --- | --- | --- | --- | --- | --- |
| Foundational services | 7 | 108 | 3.182 | 45.5 | 63.7 |
| Social infrastructure | 8 | 109 | 3.555 | 44.4 | 60.4 |
| Income | 5 | 109 | 3.371 | 67.4 | 89.0 |

Note: Principal Component Analysis is based on the correlation matrix. PC1 = first principal component. PCA is used as an exploratory benchmark. Positive first-component loadings indicate that indicators broadly move in the expected direction, while the variance explained suggests that the sub-indices remain multidimensional, as expected for formative composite measures.

**Figure A1 Rank stability under bounded random-weight simulations. Distribution of Spearman’s rho between baseline equal-weighted sub-indices and simulated sub-indices under ±25%, ±50%, and ±75% bounded weighting scenarios.**

**Table A4. Sensitivity of the Liveability Index to alternative aggregation rules**

| **Robustness check** | **Arithmetic vs geometric** | **Harmonic vs geometric** |
| --- | --- | --- |
| Spearman rank correlation | 0.9974 | 0.9934 |
| Median absolute rank shift | 1 | 1 |
| Mean absolute rank shift | 1.52 | 2.02 |
| 75th percentile absolute rank shift | 2 | 2 |
| 90th percentile absolute rank shift | 4 | 6 |
| 95th percentile absolute rank shift | 5 | 9 |
| Maximum absolute rank shift | 12 | 18 |
| Cities remaining in the same quartile | 94.50% | 96.33% |

**Note:** The geometric mean is used as the baseline specification. The arithmetic mean represents a fully compensatory benchmark, while the harmonic mean represents a more restrictive aggregation rule that penalises low-performing dimensions more strongly. Rank 1 indicates the highest level of liveability. Absolute rank shifts are computed relative to the geometric specification. Quartiles are computed separately for each index specification. N = 109 cities.

The results indicate a high degree of stability across aggregation rules. The Spearman correlations are very high in both comparisons, and the median absolute rank shift is only one position. Quartile membership is also highly stable, with 94.5% of cities remaining in the same quartile under the arithmetic specification and 96.3% under the harmonic specification. These checks suggest that the substantive ranking of cities is not driven by the chosen aggregation rule.

**Table A5. Quantile regression estimates for alternative Liveability Index specifications**

|  | **Arithmetic**  **mean** | | | **Geometric**  **mean** | | | **Harmonic**  **mean** | | |
| --- | --- | --- | --- | --- | --- | --- | --- | --- | --- |
|  | **Q25** | **Q50** | **Q75** | **Q25** | **Q50** | **Q75** | **Q25** | **Q50** | **Q75** |
| **City type** |  |  |  |  |  |  |  |  |  |
| Middle cities | Ref. | Ref. | Ref. | Ref. | Ref. | Ref. | Ref. | Ref. | Ref. |
| Other cities 45k-500k | -0.077** | -0.064** | -0.056* | -0.080*** | -0.068*** | -0.062** | -0.079*** | -0.065*** | -0.063** |
| Cities <45k | -0.053 | -0.061* | -0.045 | -0.053 | -0.068** | -0.051 | -0.051 | -0.064** | -0.069 |
| Large cities | -0.029 | -0.027 | -0.033 | -0.019 | -0.058* | -0.030 | -0.022 | -0.069** | -0.079** |
| **Geographical**  **area** |  |  |  |  |  |  |  |  |  |
| North | Ref. | Ref. | Ref. | Ref. | Ref. | Ref. | Ref. | Ref. | Ref. |
| Center | -0.101** | -0.072** | -0.061* | -0.098*** | -0.066** | -0.066** | -0.092*** | -0.074*** | -0.062* |
| South | -0.252** | -0.228** | -0.227** | -0.259*** | -0.234*** | -0.234*** | -0.251*** | -0.231*** | -0.235*** |
| Constant | 0.578** | 0.607** | 0.639** | 0.576*** | 0.608*** | 0.642*** | 0.567*** | 0.598*** | 0.638*** |
| Observations | 109 | 109 | 109 | 109 | 109 | 109 | 109 | 109 | 109 |
| Pseudo R2 | 0.488 | 0.484 | 0.420 | 0.490 | 0.483 | 0.417 | 0.486 | 0.482 | 0.399 |

**Note:** The table reports quantile regression coefficients at the 25th, 50th and 75th percentiles of the Liveability Index. The geometric mean is the baseline specification used in the main analysis. The arithmetic mean represents a fully compensatory aggregation rule, while the harmonic mean represents a more restrictive aggregation rule that penalises low-performing dimensions more strongly. Significance levels: * p < 0.10, ** p < 0.05, *** p < 0.01.

The comparison across specifications shows that the main substantive pattern is stable across aggregation rules. Other cities with populations between 45,000 and 500,000 inhabitants consistently display lower liveability scores than middle cities, and the North-South divide remains large across all specifications. The harmonic specification, which penalises low-performing dimensions more strongly, suggests a somewhat less favourable position for large cities at the median and upper quantiles.
